# Supplementary material for: Ensemble representations reveal distinct neural coding of visual working memory
Source: Nat Commun. 2019 Dec 11;10:5665. doi: 10.1038/s41467-019-13592-6 (PMC6906315; doi:10.1038/s41467-019-13592-6)
Supplement: Supplementary file 1 — Supplementary Information [file 41467_2019_13592_MOESM1_ESM.pdf]

Supplementary Discussion 1: Reconstruction with the common electrode configuration in both experiments

The different patterns of results in Experiment 1 and 2 could have been a consequence of different electrode configurations between experiments. To test this alternative explanation, we reconstructed orientation-tuning curves from a set of electrodes that were used in both experiments. The electrodes of the whole brain set were Fz, F3, F4, FC1, FC2, FC5, FC6, Cz, C3, C4, CP1, CP2, Pz, P3, P4, P7, P8, O1, and O2 (Supplementary Fig. 1a–d). The electrodes of the frontocentral areas were Fz, F3, F4, FC1, FC2, FC5, FC6, Cz, C3 (Supplementary Fig. 1e–h). The electrodes of the occipitoparietal areas were CP1, CP2, Pz, P3, P4, P7, P8, O1, and O2 (Supplementary Fig. 1i–l). Although the decoding sensitivities decreased due to the smaller number of electrodes, we found comparable results.

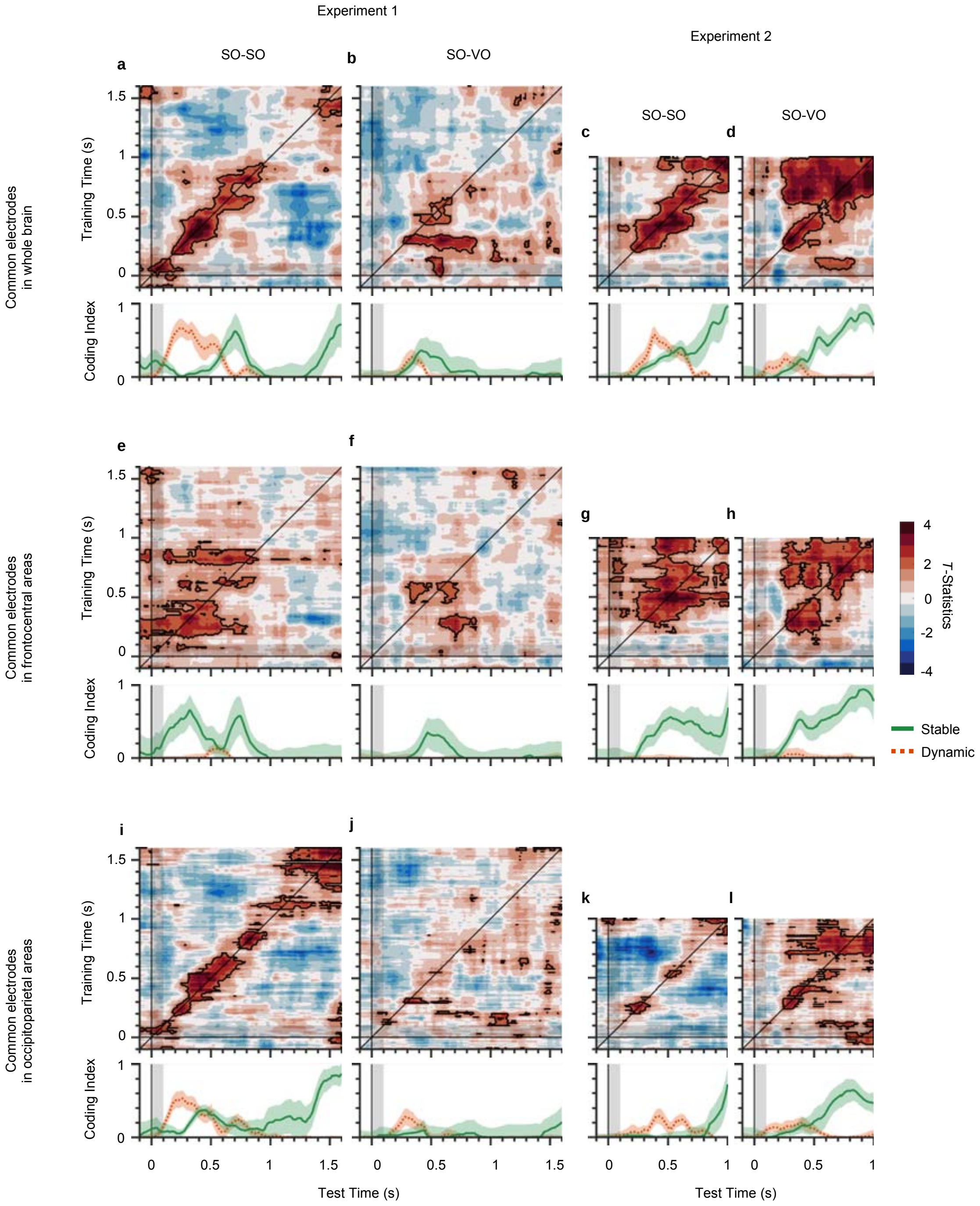

Supplementary Fig. 1. *The same results were presented from electrode configurations that were common in both experiments. Each electrode montage depicts the recordings from a set of electrodes. The x- and y-axes depict the training and generalizing time after stimulus onset, respectively. The color represents the t-statistics of the decoding sensitivity with blue representing negative results and red representing positive results.* ***a–d*** *Temporal generalization (TG) matrices of the whole brain.* ***e–h*** *TG matrices of the frontocentral areas.* ***i–l*** *TG matrices of the occipitoparietal areas*

Supplementary Discussion 2: Topographic representations of stable and dynamic coding

To investigate the detailed sources of stable and dynamic coding over time, we implemented a searchlight-based analyses and calculated a stable/dynamic index from each electrode. First, we gathered data from neighboring electrodes of each electrode from a neighborhood map, obtained using a triangulation method in FieldTrip (Oostenveld, Fries, Maris, & Schoffelen, 2011). For example, the neighbors of the Cz electrode included Fz, FC1, FC2, C3, C4, CP1, CP2, Pz, and Cz itself. Afterwards, we obtained the temporal generalization (TG) matrix of each neighbor and calculated its stable/dynamic index. The stable/dynamic index was then averaged by the 200 ms interval from 0 ms to the end of the retention interval after the stimulus onset. The stable/dynamic indices of each neighbor were aggregated and used to plot topography. The results showed that stable coding was more dominant in the frontocentral areas over a later period, whereas dynamic coding was more dominant in the occipitoparietal areas over an early period (Supplementary Fig. 2).

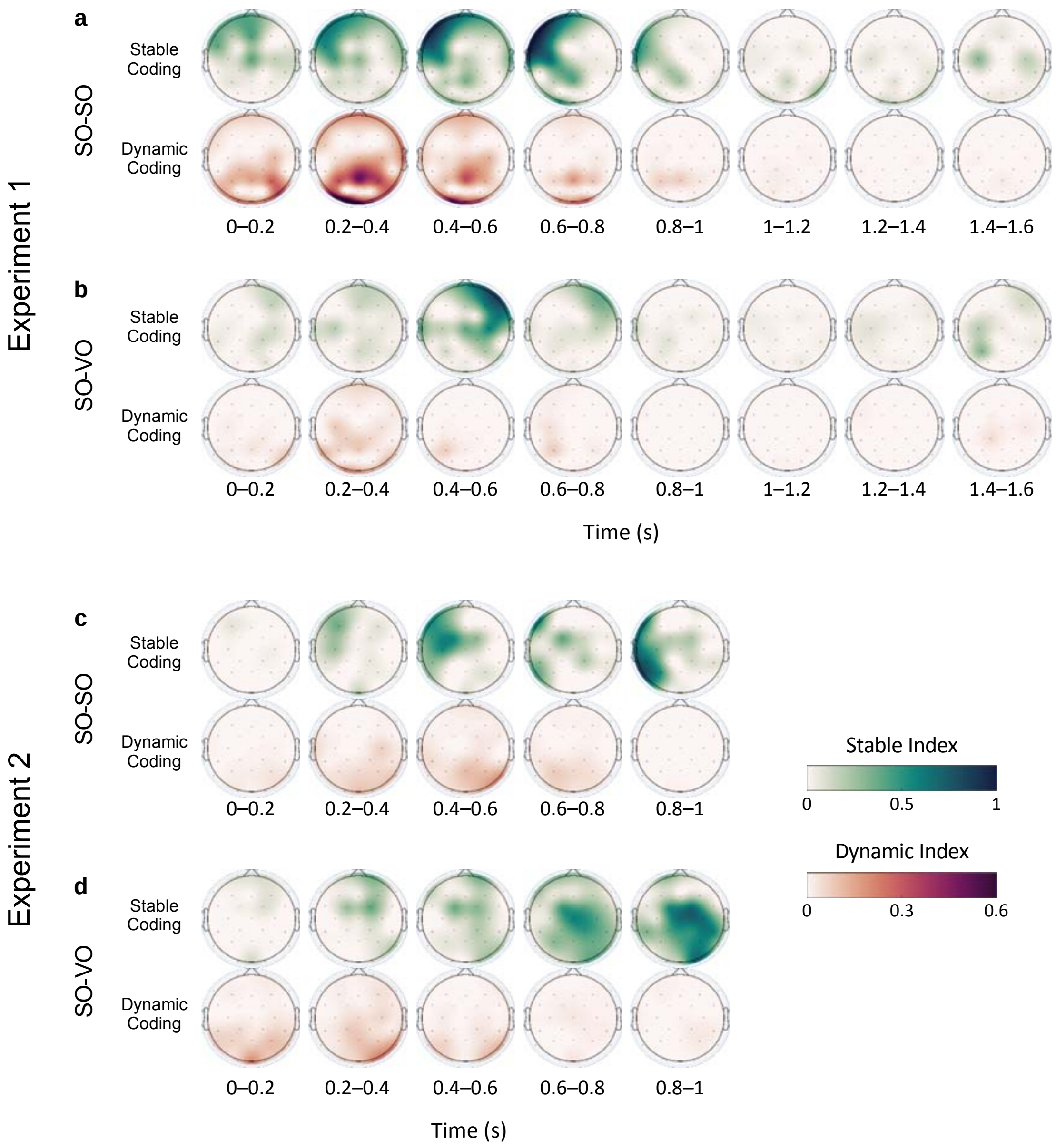

Supplementary Fig. 2. *Topographical representations of stable and dynamic coding over time.* ***a*** *Results of SO-SO prediction in Experiment 1. Full movies are provided as Supplementary Movie 1.avi and Supplementary Movie 2.avi.* ***b*** *Results of SO-VO prediction in Experiment 1. Full movies are provided as Supplementary Movie 3.avi and Supplementary Movie 4.avi.* ***c*** *Results of SO-SO prediction in Experiment 2. Full movies are provided as Supplementary Movie 5.avi and Supplementary Movie 6.avi.* ***d*** *Results of SO-VO prediction in Experiment 2. Full movies are provided as Supplementary Movie 7.avi and Supplementary Movie 8.avi. Green represents the magnitude of stable coding, and Red represents the magnitude of dynamic coding. The x-axis shows the time*

after the stimulus onset. SO – same orientation; VO – varied orientation.

Supplementary Discussion 3: Rejecting an eye movement alternative

It is important to rule out an alternative interpretation of results based on eye movements, since this can be a potential source for systematic changes in EEG for two reasons. First, eye movements can influence the EEG signal by changing the corneo-retinal dipoles (Plöchl, Ossandón, & König, 2012). Second, eye movements occur despite instructions for strict fixation in cognitive tasks (Rolfs, 2009), and can even reflect the load of visual working memory (Kang & Woodman, 2014). Further, a recent study demonstrated that the gaze positions recorded during a visual working memory task were systematically shifted depending on the remembered orientation and, not surprisingly, that remembered orientation could even be decoded from the gaze position data (Mostert et al., 2017).

Eye movements cannot explain the dynamic coding observed in the occipitoparietal electrodes. This is because eye movements, once shifted, tend to remain for an extended period of time in the absence of any stimulus; thus, they should produce a sustained impact over the retention interval. One may, nevertheless, argue that the stable coding observed at the frontocentral electrodes is susceptible to eye movements. This account is plausible. It is well known that electrodes close to the eyeballs are more susceptible to changes in the corneo-retinal dipoles (Plöchl et al., 2012). Below is a discussion relating to this topic.

We ruled out the confounding influence of eye movements based on topography. Specifically, if the impact of the eye movements are more pronounced in EEGs recorded close to the eyeballs, then the stable coding should be more pronounced if we perform the same temporal generalization analysis on the recordings from electrodes located at more anterior regions. To address this concern, we performed TG analyses of the data from the VO condition after dividing the frontocentral electrodes into further categories, using the electrode positions displayed in Supplementary Fig. 3a–d. To compare the modulations between subsets of electrodes (subset condition) and the all frontocentral electrodes (whole condition), we only isolated significant data points from these conditions. Such points are

colored green for the subset condition, red for the whole condition, and yellow for the common modulations of both conditions. If the eye movements drove the stable coding from the frontocentral electrodes, the common modulation should be more pronounced in the more anterior subset condition than in the whole condition. However, the opposite result was observed.

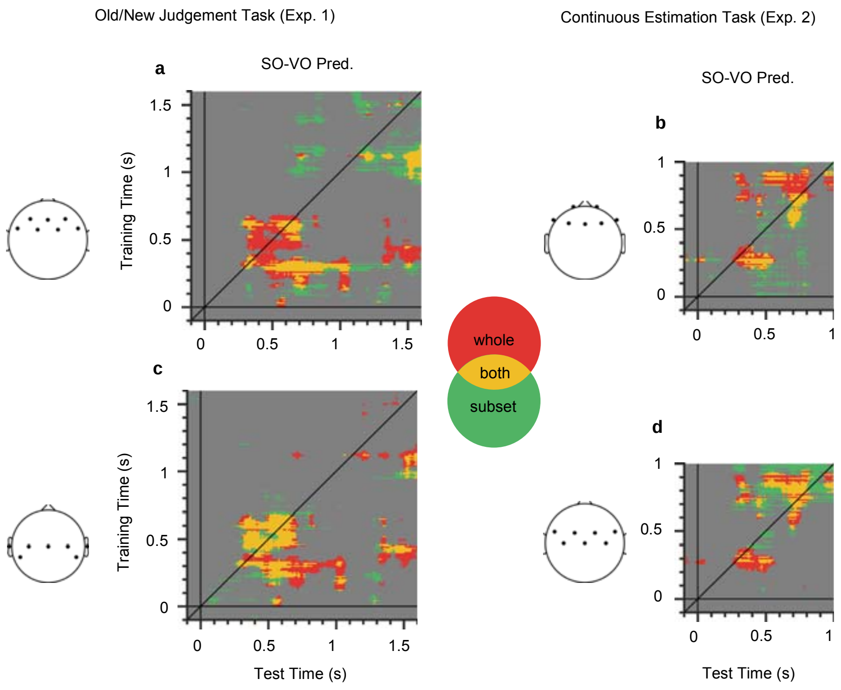

Supplementary Fig. 3. *Comparison between the entire frontocentral electrodes and a subset. Significant data points are colored green for the subset condition, red for the whole condition, and yellow for common modulations in both conditions. The electrode positions of each subset condition are illustrated topographically.*

More directly, we performed an inverted encoding model (IEM) analysis and obtained TG matrices using HEOG and VEOG signals. This was performed because the time course of decoding performance can be informative. Specifically, saccadic eye movements are suppressed immediately after any external events including visual stimulation, and this suppression tends to last approximately 300 ms (Rolfs, 2009). In addition, a recent study demonstrated that systematic eye movements do reflect the load in visual working memory,

but those movements occur more slowly than the load-dependent electrophysiological modulations (Kang & Woodman, 2014). If eye movements explain the stable coding, the time courses of stable coding should be comparable between EOGs and EEGs. Note that, because statistical power can be reduced when using only VEOG and HEOG signals, we did not emphasize statistical significance. Instead, we compared the pattern of modulations obtained from the EOG and EEG signals. We found that the meaningful modulations from the EOG signals were shifted upward by approximately 100 ms more than the EEG signals (Supplementary Fig. 4a–d). This was also true if we obtained the decoding sensitivities from the “average” and “target” response trials (Supplementary Fig. 4e–f). Consistent with the previous study, in which the occurrence of eye movements was delayed relative to the neural modulation of visual working memory (Kang & Woodman, 2014), the training set that was built from the early temporal window could not decode the remembered orientations from the EOG signals. Furthermore, if the eye movements drove the stable coding in the frontocentral electrodes, the EOG signals should also explain the behavioral index. However, as shown in Supplementary Fig. 4g–h, only insignificant correlations between the decoding sensitivities of the EOGs and the behavioral indices of ensemble representations were observed (R^2 = 0.11, p = 0.16 in Experiment 1; R^2 = 0.03, p = 0.45 in Experiment 2). Partial correlation analyses provided a consistent view. There was a significant correlation between the decoding sensitivities of the EEGs obtained from the frontocentral electrodes and the behavior while controlling the decoding sensitivities of EOGs (R^2 = 0.23, p = 0.04 in Experiment 1; R^2 = 0.25, p = 0.01 in Experiment 2). Conversely, insignificant correlations between the EOGs and behavior were observed when controlling the EEGs (R^2 = 0.09, p = 0.21 in Experiment 1; R^2 = 0.003, p = 0.81 in Experiment 2). Taken together, we conclude that our results cannot be explained by eye movements.

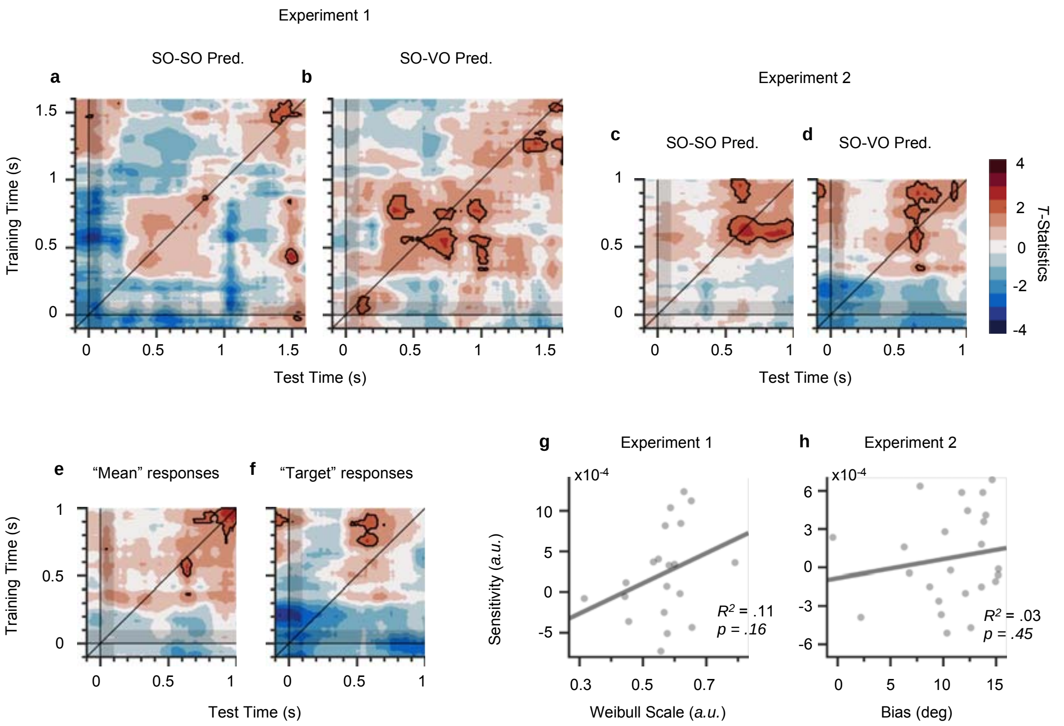

Supplementary Fig. 4. *Temporal generalization (TG) results from electroocculography (EOG) signals.* ***a–f*** *TG matrices. The x- and y-axis depict the training and generalization time after the stimulus onset, respectively. The color bar represents the t-statistics of the decoding sensitivity.* ***a–d*** *Remembered orientations and mean orientations of Experiment 1 and Experiment 2.* ***e–f*** *TG matrices of remembered mean orientations in "mean" and "target" response trials in Experiment 2.* ***g–h*** *Scatter plots between behavioral indices of ensemble representations and decoding sensitivities obtained from the EOG signals for Experiment 1 and Experiment 2. The x-axis represents each behavioral ensemble index: Weibull scale in Experiment 1 and bias in Experiment 2. The y-axis represents the summarized decoding sensitivities of the remembered mean orientations between 200–1,000 ms of training and test time.*

Supplementary Discussion 4: Robustness of across-participant correlations

We calculated the correlations between the remembered mean orientations and behavioral indices of ensemble representations in different ways to ensure that the across-participant correlations were not spurious. First, we computed non-parametric Spearman rank-order correlation coefficients which reduce the influence of outliers. We found that the

results were consistent: the correlation was significant in the frontocentral areas (*r* = .49, *p* = .049 in Experiment 1; *r* = .59, *p* = .003 in Experiment 2) but not in the occipitoparietal areas (*r* = .08, *p* = .74 in Experiment 1; *r* = −.1, *p* = .67 in Experiment 2). Second, we computed the R-squared values from the temporal windows based on the largest significant cluster across all brain regions; the window was 170–670 ms during training time and 250–870 ms during test time in Experiment 1 and 500–1,000 ms during training time and 220–1,000 ms during test time in Experiment 2. The results were still consistent: The correlation was significant in the frontocentral areas (R^2 = .21, *p* = .04 in Experiment 1; R^2 = .19, *p* = .04 in Experiment 2) but not in the occipitoparietal areas (R^2 = .0003, *p* = .94 in Experiment 1; R^2 = .05, *p* = .31 in Experiment 2). These results reaffirm the robustness of the across-participants correlations.

Supplementary Discussion 5: Time-frequency representations

Some previous studies have shown that visual working memory representations are carried by specific frequency bands in (D'Esposito & Postle, 2015; Roux & Uhlhaas, 2014). We therefore separated the EEG signals into multiple frequency bands and conducted the same analyses. However, we did not find any convincing results that a specific frequency band codes the orientation and ensemble representations.

We characterized the time-frequency representations of the orientation decoder and the ensemble representation by filtering the EEG signal into four frequency bands: theta (4–7 Hz), alpha (8–13 Hz), beta (13–30 Hz), and gamma (30–150 Hz). Based on a previous study (Foster, Bsales, Jaffe, & Awh, 2017), we obtained time-frequency representations by obtaining spectral power from Hilbert transformed data, smoothed the representation with a 100 ms moving average window and then down-sampled to 50 Hz for computational efficiency. We ran the same IEM analysis to reconstruct the remembered orientation and ensemble representation from the entire electrode array. Because we did not find any meaningful modulations, we did not analyze the data for the frontocentral and occipitoparietal areas, separately.

Specifically, in the theta band, we found several significant modulations that are

marked with closed contours. However, we did not find any consistent patterns across the two experiments in either the orientation decoder or ensemble representations (Supplementary Fig. 5a–d). In the alpha band, there appeared to be significant, stable coding in the orientation decoder during the retention interval (Supplementary Fig. 5e–h). Several previous studies have shown that the alpha band codes simple features (Bae & Luck, 2018; Wolff, Jochim, Akyürek, & Stokes, 2017), which is consistent with our results. However, we only found scattered clusters around the two-dimensional TG matrices. It is therefore difficult to conclude that alpha band codes neural representations of ensembles. We did not find any meaningful clusters in other frequency bands (Supplementary Fig. 5i–p).

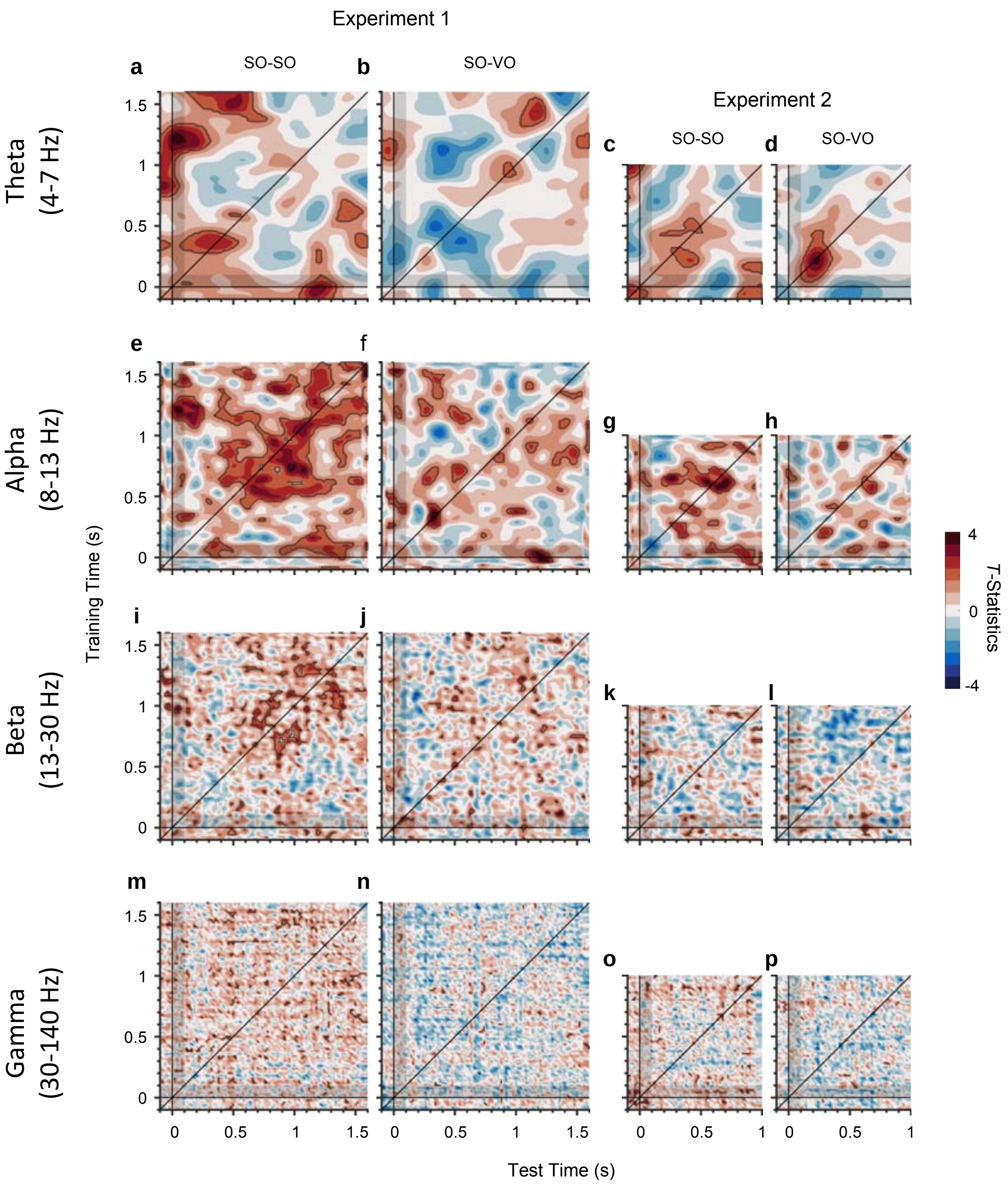

Supplementary Fig. 5. *Orientation reconstructions from total power of each frequency band. The x- and y-axes depict the training and generalizing time after stimulus onset, respectively. The color represents the t-statistics of the decoding sensitivity with blue representing negative results and red representing positive results.* ***a–f*** *Results of theta frequency band.* ***e–h*** *Results of alpha frequency band.* ***i–l*** *Results of beta frequency band.* ***m–p*** *Results of gamma frequency band.*

Supplementary References

Bae, G.-Y., & Luck, S. J. (2018). Dissociable Decoding of Spatial Attention and Working Memory from EEG Oscillations and Sustained Potentials. *The Journal of Neuroscience, 38*(2), 409–422.

D'Esposito, M., & Postle, B. R. (2015). The cognitive neuroscience of working memory. *Annual Review of Psychology*, *66*, 115–142.

Foster, J. J., Bsales, E. M., Jaffe, R. J., & Awh, E. (2017). Alpha-Band Activity Reveals Spontaneous Representations of Spatial Position in Visual Working Memory. *Current Biology*, *27*(20), 3216-3223

Kang, M.-S., & Woodman, G. F. (2014). The neurophysiological index of visual working memory maintenance is not due to load dependent eye movements. *Neuropsychologia*, *56*, 63–72.

Mostert, P., Albers, A. M., Brinkman, L., Todorova, L., Kok, P., & Lange, F. P. de. (2018). Eye movement-related confounds in neural decoding of visual working memory representations. *eNeuro.* ENEURO.0401-17.2018

Oostenveld, R., Fries, P., Maris, E., & Schoffelen, J.-M. (2011). FieldTrip: Open source software for advanced analysis of MEG, EEG, and invasive electrophysiological data. *Computational Intelligence and Neuroscience*, *2011*, 156869.

Plöchl, M., Ossandón, J. P., & König, P. (2012). Combining EEG and eye tracking: identification, characterization, and correction of eye movement artifacts in electroencephalographic data. *Frontiers in Human Neuroscience*, *6*, 1–23.

Rolfs, M. (2009). Microsaccades: small steps on a long way. *Vision Research*, *49*(20), 2415–2441.

Roux, F., & Uhlhaas, P. J. (2014). Working memory and neural oscillations: Alpha-gamma versus theta-gamma codes for distinct WM information? *Trends in Cognitive Sciences*, *18*(1), 16–25.

Wolff, M. J., Jochim, J., Akyürek, E. G., & Stokes, M. G. (2017). Dynamic hidden states underlying working-memory-guided behavior. *Nature Neuroscience*, *20*(6). 864-871
