# Supplementary material for: Ensemble representations reveal distinct neural coding of visual working memory
Source: Nat Commun. 2019 Dec 11;10:5665. doi: 10.1038/s41467-019-13592-6 (PMC6906315; doi:10.1038/s41467-019-13592-6)
Supplement: Supplementary file 3 — Reporting Summary [file 41467_2019_13592_MOESM3_ESM.pdf]

natureresearch

Corresponding author(s): Min-Suk Kang

Last updated by author(s): Sep 27, 2019

Reporting Summary

Nature Research wishes to improve the reproducibility of the work that we publish. This form provides structure for consistency and transparency in reporting. For further information on Nature Research policies, seeAuthors & Referees and theEditorial Policy Checklist .

Statistics

For all statistical analyses, confirm that the following items are present in the figure legend, table legend, main text, or Methods section.

n/a	Confirmed	
☐	☒	The exact sample size (*n*) for each experimental group/condition, given as a discrete number and unit of measurement
☐	☒	A statement on whether measurements were taken from distinct samples or whether the same sample was measured repeatedly
☐	☒	The statistical test(s) used AND whether they are one- or two-sided<br>*Only common tests should be described solely by name; describe more complex techniques in the Methods section.*
☐	☒	A description of all covariates tested
☐	☒	A description of any assumptions or corrections, such as tests of normality and adjustment for multiple comparisons
☐	☒	A full description of the statistical parameters including central tendency (e.g. means) or other basic estimates (e.g. regression coefficient) AND variation (e.g. standard deviation) or associated estimates of uncertainty (e.g. confidence intervals)
☐	☒	For null hypothesis testing, the test statistic (e.g. *F*, *t*, *r*) with confidence intervals, effect sizes, degrees of freedom and *P* value noted<br>*Give P values as exact values whenever suitable.*
☒	☐	For Bayesian analysis, information on the choice of priors and Markov chain Monte Carlo settings
☒	☐	For hierarchical and complex designs, identification of the appropriate level for tests and full reporting of outcomes
☒	☐	Estimates of effect sizes (e.g. Cohen's *d*, Pearson's *r*), indicating how they were calculated

Our web collection on statistics for biologists contains articles on many of the points above.

Software and code

Policy information about availability of computer code

Data collection	Matlab 2016b - Commercial Products from MathWorks<br>BrainVision Recorder - Commercial Products from Brain Products GmbH.<br>Psychophysical Toolbox 3 - An open Matlab toolbox
Data analysis	Matlab 2016b with Signal Processing, Image Processing, Statistical Processing and Parallel Processing Toolboxes- Commercial Products from MathWorks<br>R - An open source statistical software for statistical analysis<br>EEGLab toolbox, CircStat toolbox, and several files (c.g. cbrewer) that are available through File Exchange.<br>Custom scripts

For manuscripts utilizing custom algorithms or software that are central to the research but not yet described in published literature, software must be made available to editors/reviewers. We strongly encourage code deposition in a community repository (e.g. GitHub). See the Nature Research guidelines for submitting code & software for further information.

Data

Policy information about availability of data

All manuscripts must include a data availability statement. This statement should provide the following information, where applicable:

- Accession codes, unique identifiers, or web links for publicly available datasets
- A list of figures that have associated raw data
- A description of any restrictions on data availability

We make all our data and analysis files available upon publication.

Field-specific reporting

Please select the one below that is the best fit for your research. If you are not sure, read the appropriate sections before making your selection.

☐ Life sciences ☒ Behavioural & social sciences ☐ Ecological, evolutionary & environmental sciences

For a reference copy of the document with all sections, see nature.com/documents/nr-reporting-summary-flat.pdf

Behavioural & social sciences study design

All studies must disclose on these points even when the disclosure is negative.

Study description	We conducted an old/new judgment task in Experiment 1 and a continuous estimation task in Experiment 2. Both tasks were computer based behavioral task, resulting in discrete correct and incorrect responses and continuous response errors. EEG data were collected simultaneously.
Research sample	Twenty-seven participants including the third author (M = 24 years, SD = 4.15, 12 females) and 35 participants (M = 23.03 years, SD = 2.5, 20 females) were recruited for Experiment 1 and 2, respectively. All participants reported normal or corrected-to-normal vision.
Sampling strategy	The sample size was closely matched to previous studies that used similar methods, Studies applying inverted encoding models to EEG data.
Data collection	Stimuli were generated and controlled using a Mac Mini, MATLAB, and the Psychophysics Toolbox. The stimuli were presented on a CRT monitor with a refresh rate of 100 Hz and a resolution of 1,024 by 768 pixels at a viewing distance of ~85 cm. The EEG signal was recorded at 500 Hz using 32 Ag/AgCl electrodes mounted in an elastic cap and amplified by an ActiCHamp amplifier (BrainVision). The signal was low-pass filtered (140 Hz) online. The impedance of all electrodes was kept below 10 kΩ throughout the experiment.
Timing	We ran Experiment 1 from 2017-09-18 to 2018-01-26 and Experiment 2 from 2018-03-13 to 2018-06-14.
Data exclusions	Trials with artifacts (e.g. blinks, eye movements, and drift) were identified and excluded based on visual inspection according to the standard procedures (Luck, 2014).
Non-participation	Seven participants were excluded from the analysis of Experiment 1 (one participant did not complete the experiment, and six participants had less than 80% artifact-free trials). Eleven participants were excluded from the analysis of Experiment 2 (four participants did not complete the experiment due to excessive EEG noises, six participants had less than 80% artifact-free trials, and one participant had to stop, due to a recording failure).
Randomization	The study was a within-subject design where participants performed all types of conditions.

Reporting for specific materials, systems and methods

We require information from authors about some types of materials, experimental systems and methods used in many studies. Here, indicate whether each material, system or method listed is relevant to your study. If you are not sure if a list item applies to your research, read the appropriate section before selecting a response.

Materials & experimental systems

n/a	Involved in the study	
☒	☐	Antibodies
☒	☐	Eukaryotic cell lines
☒	☐	Palaeontology
☒	☐	Animals and other organisms
☐	☒	Human research participants
☒	☐	Clinical data

Methods

n/a	Involved in the study	
☒	☐	ChIP-seq
☒	☐	Flow cytometry
☒	☐	MRI-based neuroimaging

Human research participants

Policy information about studies involving human research participants

Population characteristics	See above
Recruitment	We recruited participants from the paid subject pool with a condition that they should at least have participated in one computer based experimental study. The paid subject pool has been built by advertising experimental studies over the announcement board run by Sungkyunkwan University.
Ethics oversight	Sungkyunkwan University Institutional Review Board

Note that full information on the approval of the study protocol must also be provided in the manuscript.
