# Supplementary material for: Ensemble representations reveal distinct neural coding of visual working memory
Source: Nat Commun. 2019 Dec 11;10:5665. doi: 10.1038/s41467-019-13592-6 (PMC6906315; doi:10.1038/s41467-019-13592-6)
Supplement: Supplementary file 4 — Description of Additional Supplementary Files [file 41467_2019_13592_MOESM4_ESM.pdf]

Description of Additional Supplementary Files

File Name: Supplementary Movie 1
Description: Topographical representations of dynamic coding of SO-SO prediction in Experiment 1 are drawn over time.

File Name: Supplementary Movie 2
Description: Topographical representations of stable coding of SO-SO prediction in Experiment 1 are drawn over time.

File Name: Supplementary Movie 3
Description: Topographical representations of dynamic coding of SO-VO prediction in Experiment 1 are drawn over time.

File Name: Supplementary Movie 4
Description: Topographical representations of stable coding of SO-VO prediction in Experiment 1 are drawn over time.

File Name: Supplementary Movie 5
Description: Topographical representations of dynamic coding of SO-SO prediction in Experiment 2 are drawn over time.

File Name: Supplementary Movie 6
Description: Topographical representations of stable coding of SO-SO prediction in Experiment 2 are drawn over time.

File Name: Supplementary Movie 7
Description: Topographical representations of dynamic coding of SO-VO prediction in Experiment 2 are drawn over time.

File Name: Supplementary Movie 8
Description: Topographical representations of stable coding of SO-VO prediction in Experiment 2 are drawn over time.
